# Marching Cubes and Histogram Pyramids for 3D Medical Visualization

**DOI:** 10.3390/jimaging6090088

**Published:** 2020-09-03

**Authors:** Porawat Visutsak

**Affiliations:** Department of Computer and Information Science, Faculty of Applied Science, King Mongkut’s University of Technology North Bangkok, Bangkok 10800, Thailand; porawatv@kmutnb.ac.th; Tel.: +66-2555-2000 (ext. 4225)

**Keywords:** marching cubes, histogram pyramids, volumetric rendering, 3D medical model, smooth voxel, isosurface

## Abstract

This paper aims to implement histogram pyramids with marching cubes method for 3D medical volumetric rendering. The histogram pyramids are used for feature extraction by segmenting the image into the hierarchical order like the pyramid shape. The histogram pyramids can decrease the number of sparse matrixes that will occur during voxel manipulation. The important feature of the histogram pyramids is the direction of segments in the image. Then this feature will be used for connecting pixels (2D) to form up voxel (3D) during marching cubes implementation. The proposed method is fast and easy to implement and it also produces a smooth result (compared to the traditional marching cubes technique). The experimental results show the time consuming for generating 3D model can be reduced by 15.59% in average. The paper also shows the comparison between the surface rendering using the traditional marching cubes and the marching cubes with histogram pyramids. Therefore, for the volumetric rendering such as 3D medical models and terrains where a large number of lookups in 3D grids are performed, this method is a particularly good choice for generating the smooth surface of 3D object.

## 1. Introduction

**Figure 1:**
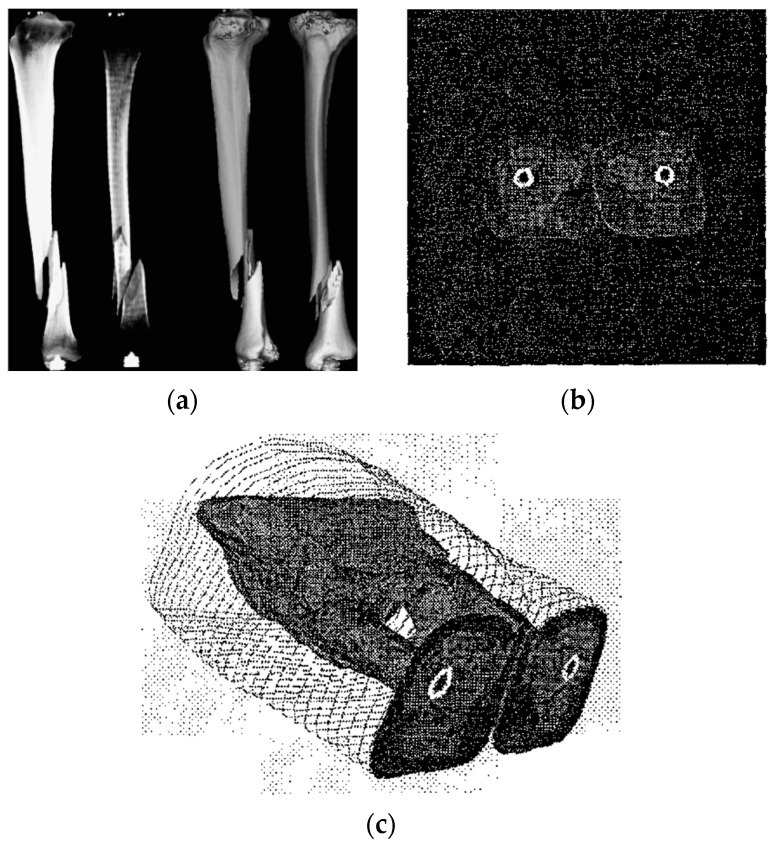
(**a**) Bone fracture displays in 3D visualization software, the Sobel-filter is used for detecting the outline of bone [11]; (**b**) CT image slides and (**c**) 3D visualization [4].

**Figure 2:**
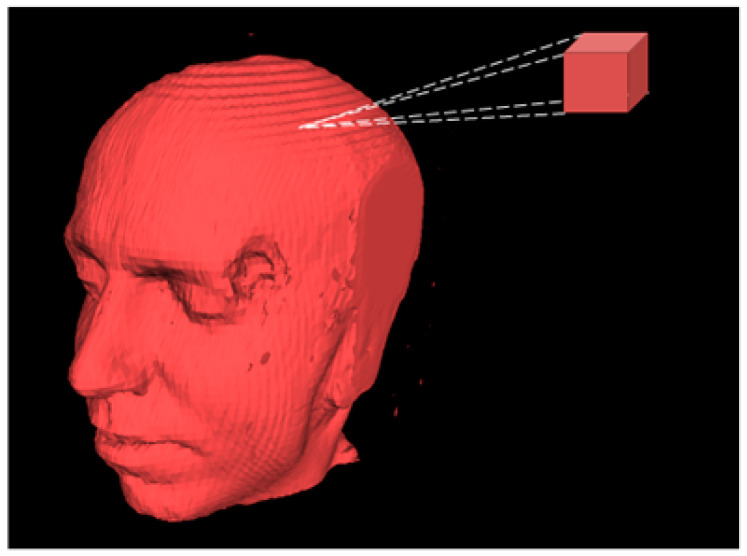
3D human head model, constructed by marching cubes method (the original source file of this figure is [12]).

3D visualization methods of human bone and organs were applied to diagnosis as long as 100 years ago [1]. Normally, 3D medical imaging system aims to provide both quantitative and qualitative information for diagnosis. 3D visualization system can be divided into 4 operations: (1) preprocessing that deals with the volume of interest and features extraction; (2) visualization processes generate 3D object from 2D images; (3) manipulation explains the geometry of object that can be distorted and deformed; (4) analysis that deals with methods of quantify 3D object [2]. For medical volumetric rendering where 2D binary images (CT-volume) are feed to construct 3D object, collision detection algorithm is used to manipulate the intersecting fragments and generate triangulated mesh models (see Figure 1a). The doctor can take this advantage of training by observing the demonstration of context of 3D manipulation of bone fragments and the resulting CT images [3]. More works of 3D visualization such as texture mapping and semi-automatic image segmentation. Texture mapping technique constructs 3D object by interpreting the spatial relationships of 2D binary images and generates 3D visualization of perspective-mapped from each image layer [4]. Whereas, semi-automatic image segmentation allows the doctor to make the segmentation of subject by the area of interest [5]. Therefore, the major drawback of these methods is the computational cost of real time resampling for making texture in 3D object reconstruction process [4], and infeasibility for each individual segmentation [5] (see Figure 1b,c). The alternative choice for 3D volumetric rendering is marching cubes. The marching cubes method keeps the coordinates conveyed by traversing the outline of 2D binary shape and marches them to construct 3D object [6,7,8,9,10]. The algorithm is based on the configuration of 15 fundamental cubes (see Section 2). Figure 2 shows 3D human head model constructed by marching cubes method; the method used 150 slides of 2D image as input source.

It seems like the marching cubes can reduce the computational time used for resampling in 3D reconstruction, but the problem still remains in observing the qualitative information of 3D surface constructed using marching cubes. One major problem of marching cubes is the unused voxels which can be generated during parsing the coordinates and the intensity values of 2D images, these unused voxels affect for the smoothness of 3D surface (the detail of the surface smoothness will be explained in Section 4 and Section 6). To overcome this drawback, this paper introduces histogram pyramids with marching cubes method for 3D medical volumetric rendering. The histogram pyramids organize the image entries to form the voxel related to the index values, the traversal of histogram pyramids is used to construct the point list that will be later used for generating the voxel. The organization of paper consists of (1) Introduction, (2) The brief concept of marching cubes method, (3) Reading the intensity value of CT images for 3D rendering, (4) Histogram pyramids, (5) Implementation, (6) Results, and (7) Conclusion and future works.

## 2. Marching Cubes

Original marching cubes algorithm was developed by [6], later works applied this algorithm for 3D rendering using 2D medical images (CT: Computed Tomography Scan, MRI: Magnetic Resonance Imaging, and SPECT: Single-Photon Emission Computed Tomography) sequence as an input. The marching cubes algorithm will be explained later in this section. More works of volume rendering techniques such as [7] used sampled scalar functions for displaying 3D surfaces, [8] presented the extended version of volume rendering that used to handle images with mixtures properties (color and texture). To deal with the boundary value problems which are the main task for detecting the outline of 2D binary shape, [9] applied the shortest path analysis to the speed function and used it to solve an unknown in the surface function. [10] used the endoscope images for 3D surface reconstruction; the sophisticated equipment was used as the fine-tuned measurement of object together with 3D grid that would be fit for the specific endoscope image.

**Figure 3:**
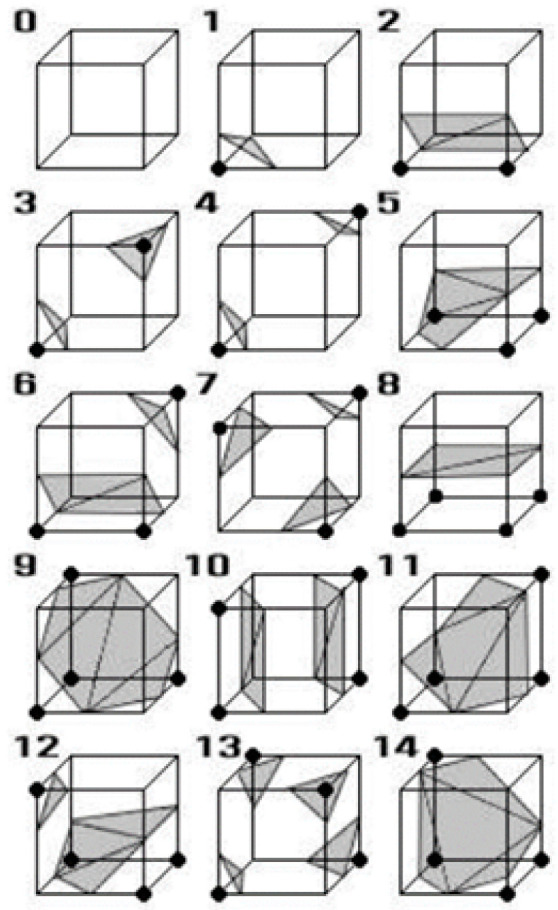
The configuration of marching cubes based on [6].

As mentioned earlier, marching cubes algorithm is based on the configuration of 15 fundamental cubes [6]. 2D medical images (slides) contained the scalar value of gray scale will be used to construct the cubes based on their indexes correspond to the configuration of 15 fundamental cubes as shown in Figure 3. Based on [13,14], a cube consists of eight vertices that can contain two values of 0 and 1; note that the value of 0 implies that there is no information conveyed in the vertex (the vertex will not be used to form the next cube for 3D object rendering), whereas the value of 1 implies that the vertex contains the information and will be used to configure the next cube as the connecting vertex.

**Figure 4:**
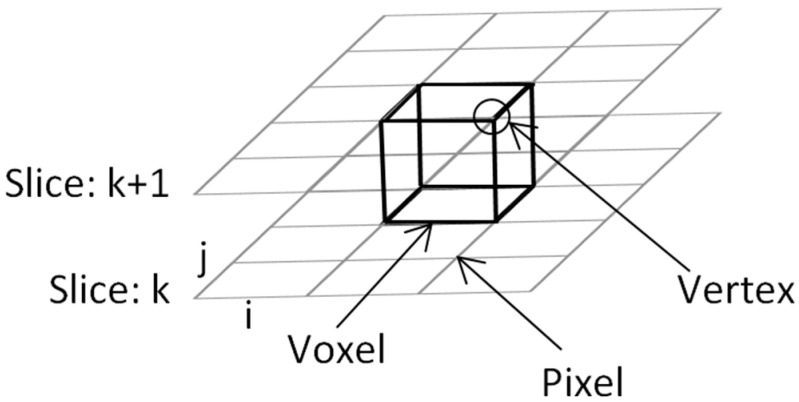
The connection of vertices to form the cube.

The first step of vertex configuration to form the cube begins with connecting the upper slice; the first four vertices locate in lower slice and the second four vertices will be in the upper one as illustrated in Figure 4. Next, the algorithm will determine the inner cut within the cube by using these criterions:

- The vertex will be the inner vertex if the vertex value is greater than or equal to the isosurface value; then assign “0” to the vertex.
- The vertex will be the outer vertex if the vertex value is less than the isosurface value; then assign “1” to the vertex.

Therefore, they will have 2^8^ = 256 combinations per one cube (2 = two values of 0, 1; 8 = number of vertices in 1 cube).

**Figure 5:**
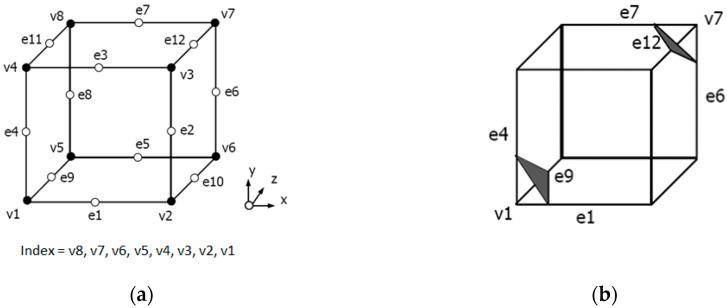
(**a**) The configuration of index; (**b**) The example of index contains value “01000001” and the triangular cross-section according to index value.

**Figure 6:**
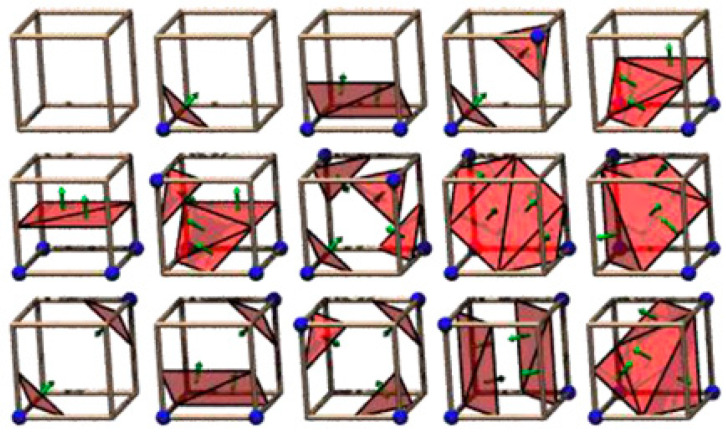
The direction of the triangular cross-section determined by normal vector [15].

The third step of marching cubes is the creating of index; the index will be used to match the configuration of 15 fundamental cubes in Figure 3. Figure 5a shows the configuration of index and Figure 5b shows the example of index that contains the value = 01000001, this index matches the cube no. 4 in Figure 3. It means that the cube will be sheared off at edge no. 1, 4, 9 (e1, e4, e9) and edge no. 6, 7, 12 (e6, e7, e12), respectively. In the last step of marching cubes, the algorithm will determine the direction of the cut using normal vector (see Figure 6).

## 3. Reading the Intensity Value of CT Images for 3D Rendering

**Figure 7:**
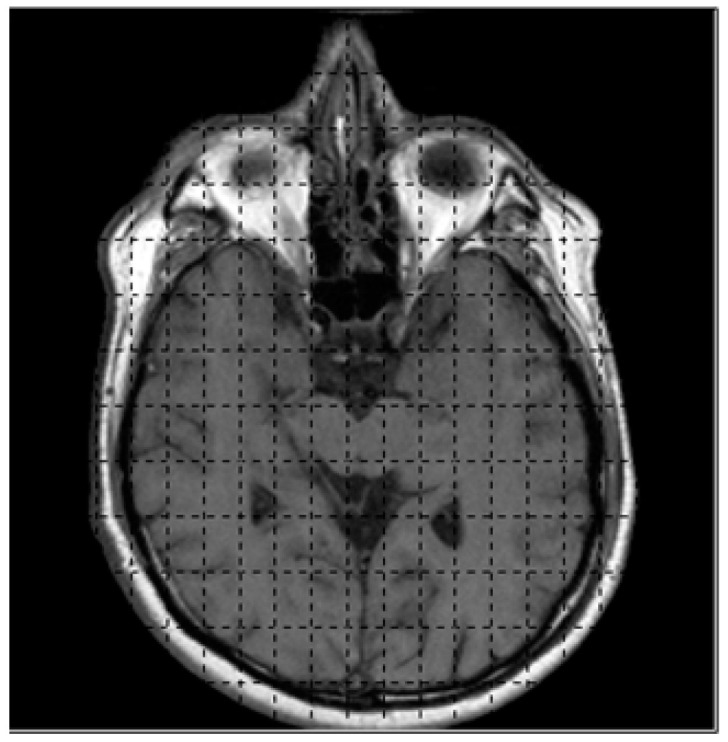
The input image with 128 × 128 segments.

The first step for obtaining the intensity value of medical images begins with reading the coordinates and values then store them in the data structure of the system, e.g., the binary image with dimension of 256 × 256 will be read and kept in the array. The input array will be manipulated for marching cubes and histogram pyramids. Figure 7 shows the binary image and grid structure used for dividing image into 128 × 128 segments.

**Figure 8:**
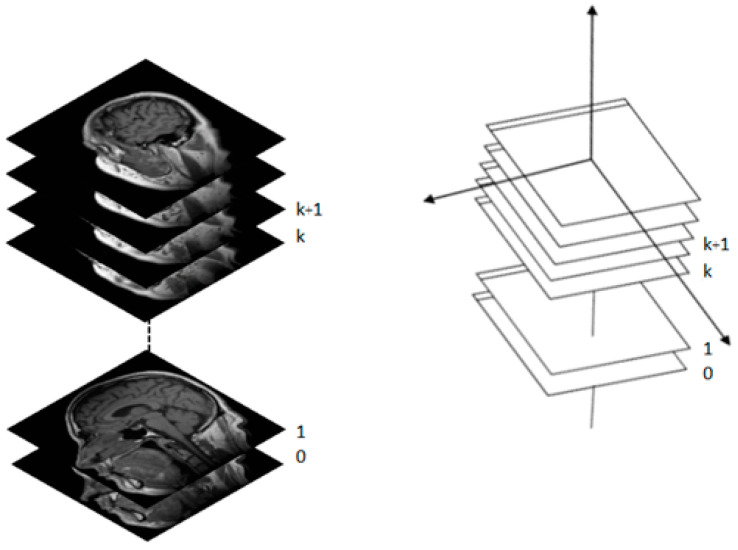
The CT images sequence used in 3D medical rendering.

The marching cubes algorithm will generate 3D visualization of 2D binary images by manipulating in form of width x height of image; e.g., in MRI, the resolution is determined by the number of pixels in a specified FOV (Field-of-view), for FOV = 320, the input image with dimension of 320 × 320 will obtain the amount of data = (320 + 1) × (320 + 1) = 103,041; these data will be stored in the array [0, …, 103,040]. The data that will be kept in the array is the intensity value of input image; in the gray-scale image, the range of gray-scale values are varying from 0 to 255 (256 intensity values). The rendering process will be computed starting from the first slide until the last slide of images (*k*, …, *k* + 1) as illustrated previously in Figure 4. Figure 8 shows the CT images sequence.

## 4. Histogram Pyramids

**Figure 9:**
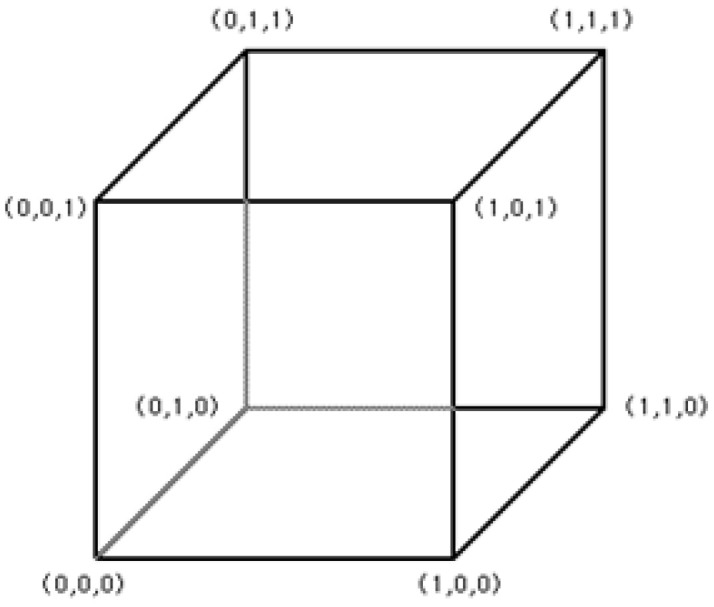
The vertices of cubes used for creating the histogram pyramids index.

One major problem of manipulating the large amount of image data is sparse matrix. The main idea of histogram pyramids is used for preventing sparse matrix [16,17,18]. The sparse matrix can be occurred during the process of connecting image slides, many elements in sparse matrix can be spread out and most of them will not be used for generating the voxel. Figure 9 shows the index of histogram pyramids created from vertices, e.g., V000 → 0, V100 → 1, V010 → 2, V001 → 3, V101 → 4, V011 → 5, V110 → 6, V111 → 7, and the last index (index no. 8) will be kept as the starting point of the traversal of histogram pyramids.

**Figure 10:**
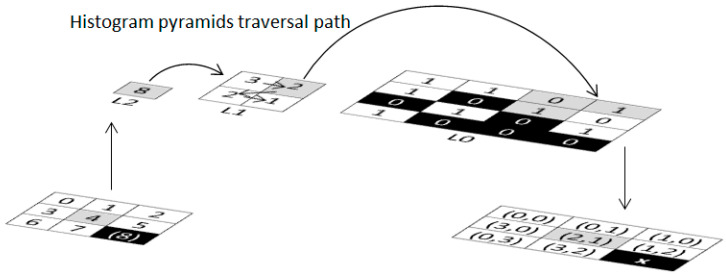
Histogram pyramids traversal.

If V101 (index no. 4) contains maximum intensity value, the histogram pyramids method can be traversed as shown in Figure 10. In this example, a 2D array contains nine elements (the index range = [0…8]) used to represent nine entries of 2D image. Note that *x* will be terminated when the summation of index pointer exceeds the maximum value of the index range (8). In the example in Figure 10, the traversal path is started with index no. 4, and L2 will be set to 0, 8 (0 + 8 ≥ 8; thus, this point is fit). The list will be lower down to L1 and retain start = 0. In L1: the upper left element, the index range will be set to 0, 3; the upper right element, the index range will be set to 3, 5 (3 + 5 ≥ 8; thus, this point is fit). The list will be lower down to L0 and retain start = 3. In L0: the upper left element = empty; the upper right element, the index range will be set to 3, 4; the lower left element, the index range will be set to 4, 5 (4 + 5 ≥ 8; this point is fit). The resulting point = (2, 1). The resulting point list will be used later in marching cubes process.

## 5. Implementation

**Figure 11:**
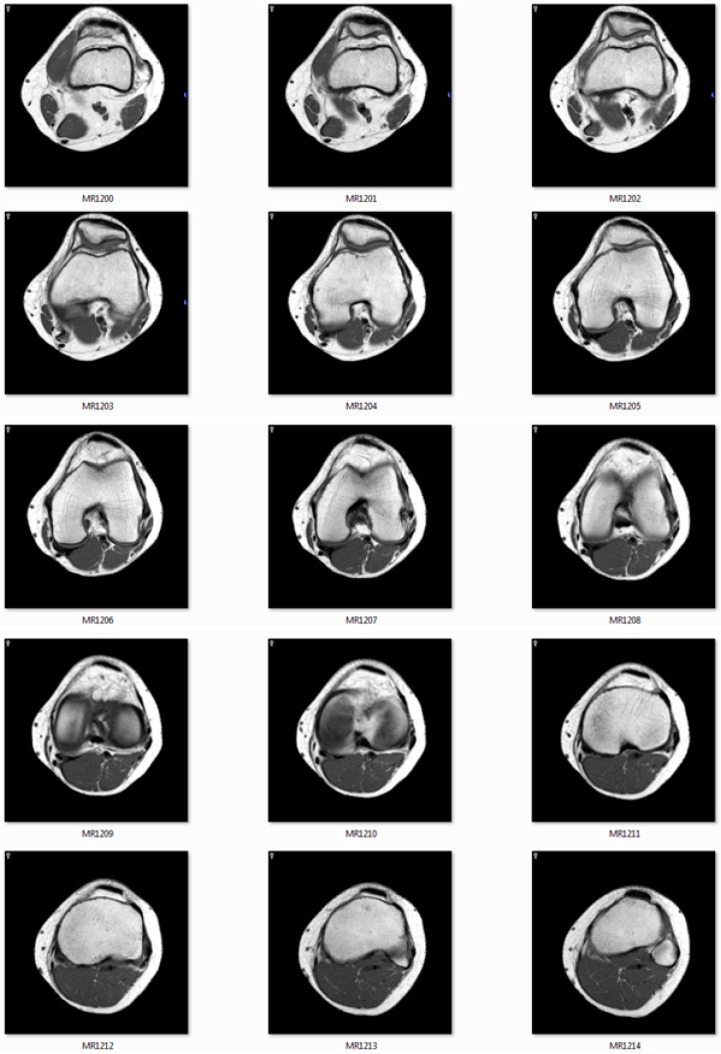

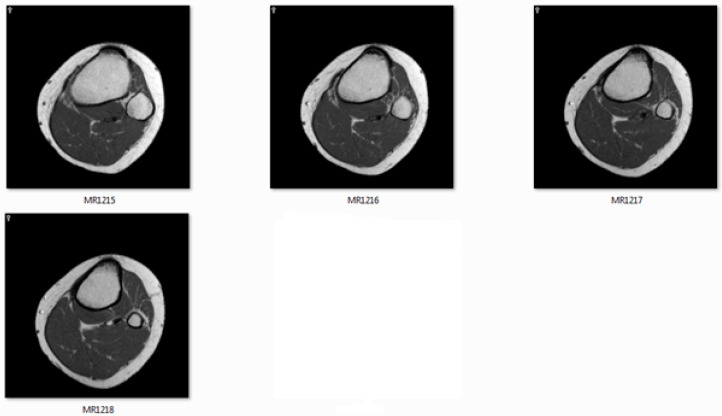
The image slides used as input data.

The CT/MRI of 32/64/128 slides must be provided to use as input images. Figure 11 shows the MRI slides of human knee.

**Figure 12:**
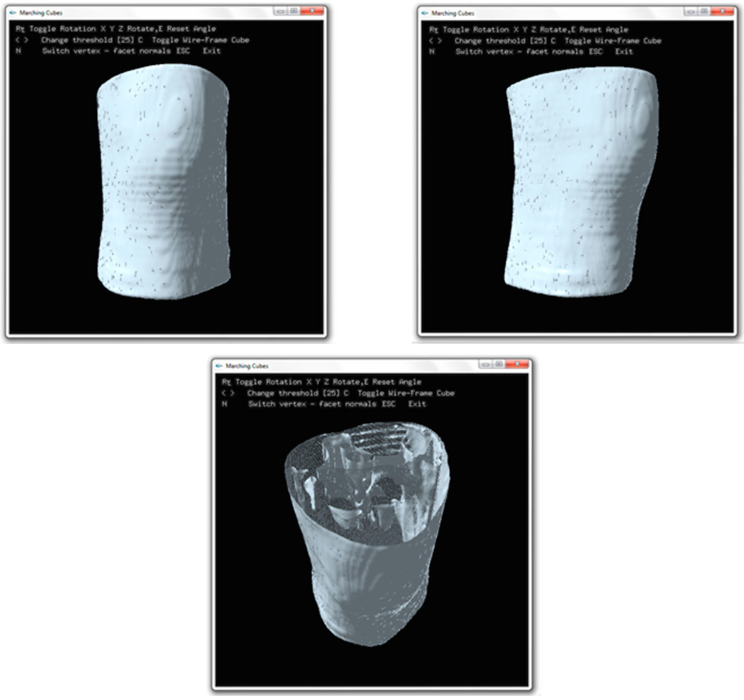
The screen captured of 3D medical visualization program.

Two important parameters will be read: the coordinates and the intensity values; the point list of histogram pyramids will be computed for marching cubes process. The user interface of the program is shown in Figure 12, the program may prompt to the user for rotate the result 3D object and toggle to the wireframe mode. The screen captured of the program is also shown in Figure 12.

## 6. Results

**Figure 13:**
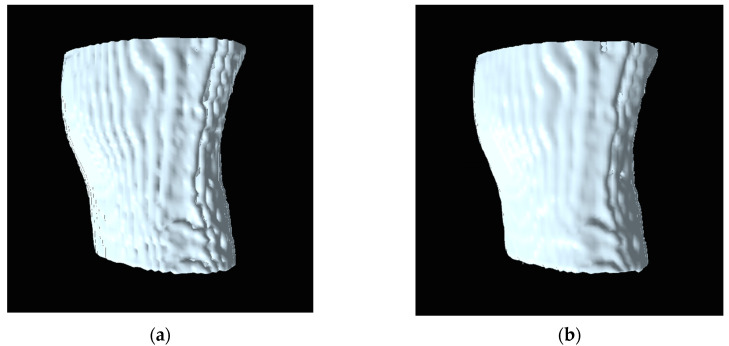
(**a**) The surface rendering using the traditional marching cubes [14]; (**b**) the surface rendering using the marching cubes with histogram pyramids.

As mentioned earlier in Section 4, the histogram pyramids can reduce the redundant voxels which are not used for generating 3D surface. These unused voxels affect for causes of surface roughness in 3D reconstruction. Figure 13 shows the comparison of surface rendering between the marching cubes [14] and the marching cubes with histogram pyramids, by reducing the unused voxel, the marching cubes with histogram pyramids can generate the smooth surface. To measure the smoothness of surface, [19] proposed the edge point computation and local window to calculate the distance between the vertices. The average curvature value is calculated for each vertex, and it corresponds to the mean of the curvature of all vertices from its local window. The curvature value is the parameter to measure the roughness of 3D mesh model [19]. Generally, the common parameter used to determine the curvature is mesh density (vertices). In [19,20], the work already proved that a higher mesh density associated to the roughness of 3D mesh model. Therefore, the model generated using the traditional marching cubes will have higher mesh density than the model generated using the marching cubes with histogram pyramids. Table 1 shows the comparison of mesh densities between the marching cubes [14] and the marching cubes with histogram pyramids.

**Figure 14:**
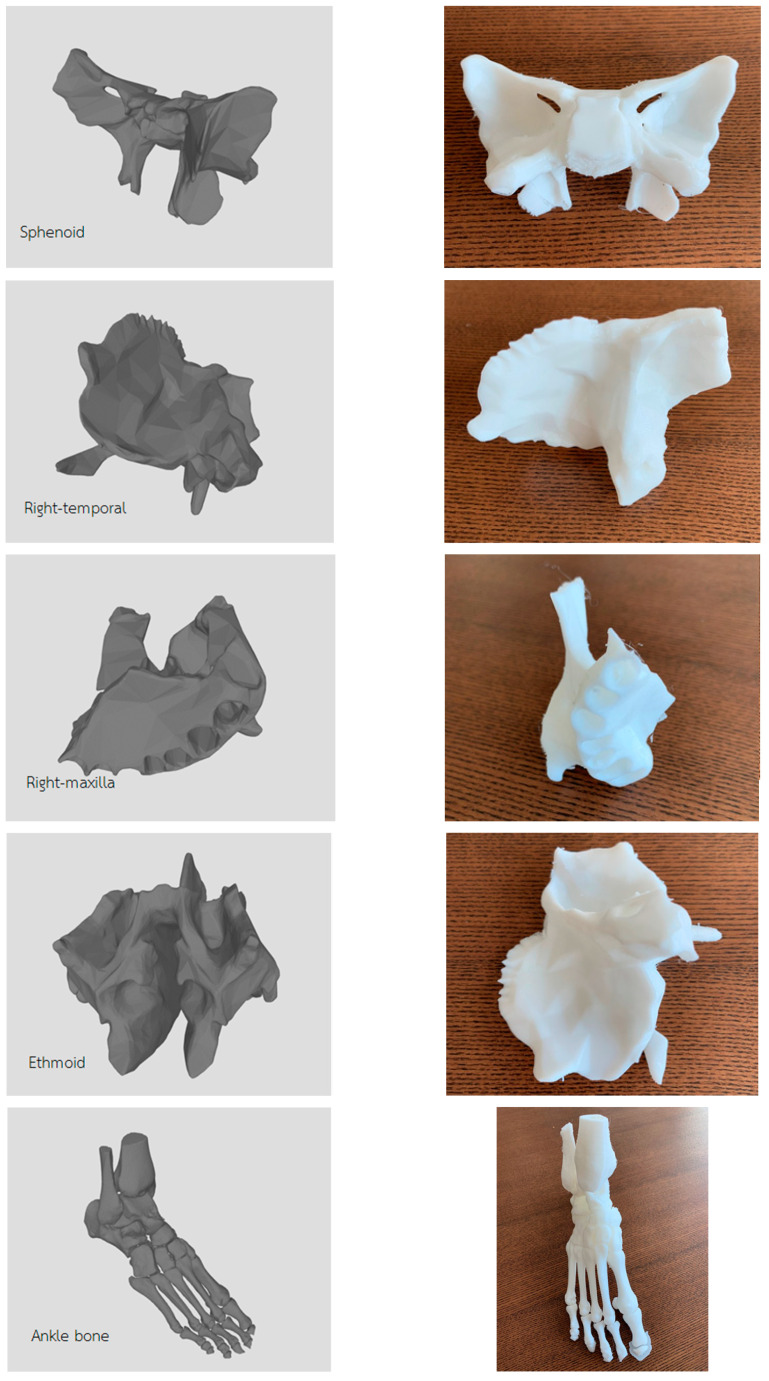

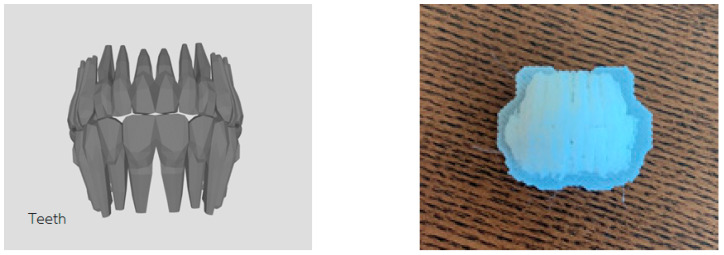
3D rendering results and their 3D printing.

The system was tested using six models; Table 1 shows the results of experiment, the rendering times of marching cubes compared with the rendering times of marching cubes with histogram pyramids. The paper also tested models by printing them with 3D printer using printing resolution = 200 µm. The results of rendering models and their 3D printings are shown in Figure 14.

## 7. Conclusions and Future Works

This paper presents the method for 3D visualization using marching cubes and histogram pyramids. The input image slides of CT/MRI were fed into the system; two important parameters, coordinates and intensity values of image, were computed using histogram pyramids to generate point list; the system later used point list for marching cubes process. The relationships of voxel among the layers of medical images (*k* and *k* + 1) were manipulated to create the sequence of cubes and form up 3D object. The paper also shows the comparison between the rendering times of marching cubes and the rendering times of marching cubes with histogram pyramids. The paper also shows the smoothness of 3D object generated from the marching cubes with histogram pyramids. The results show that the mesh density is the major factor affecting the experiment. Table 1 shows the time consuming for generating 3D model can be reduced by 15.59% in average. The complexity of algorithm of histogram pyramids for reducing sparse matrix gives O (N) + M (log N), where N is number of elements and M is number of lists. The recommendations of future works such as improve the histogram pyramids algorithm and conduct the research using terrain data in 3D computer game.

## Figures and Tables

**Table 1:** The experimental results.

Model	Mesh Density (Vertices)		Rendering Times (Sec.)		% Times Reduced
	Marching Cubes [14]	Marching Cubes with Histogram Pyramids	Marching Cubes [14]	Marching Cubes with Histogram Pyramids	
Sphenoid	45,440	38,800	0.345	0.295	14.49
Right-temporal	39,600	32,200	0.338	0.277	18.04
Right-maxilla	38,560	31,900	0.324	0.268	17.28
Ethmoid	49,800	43,800	0.382	0.333	12.82
Ankle bone	63,120	52,900	0.595	0.500	15.96
Teeth	69,760	59,200	0.467	0.397	14.98
-	-	-	-	Average	15.59
